# ABO blood groups as a prognostic factor for recurrence in ovarian and vulvar cancer

**DOI:** 10.1371/journal.pone.0195213

**Published:** 2018-03-29

**Authors:** Céline Montavon Sartorius, Andreas Schoetzau, Henriette Kettelhack, Daniel Fink, Neville F. Hacker, André Fedier, Francis Jacob, Viola Heinzelmann-Schwarz

**Affiliations:** 1 Department of Gynecology and Gynecological Oncology, Hospital for Women, University Hospital Basel and University of Basel, Basel, Switzerland; 2 Ovarian Cancer Research, Department of Biomedicine, University Hospital Basel and University of Basel, Basel, Switzerland; 3 Department of Gynecology, University Hospital Zurich and University of Zurich, Zurich, Switzerland; 4 Gynaecological Cancer Center, Royal Hospital for Women, School of Women’s and Children’s Health, UNSW, Sydney, Australia; 5 Glyco-Oncology Research, Department of Biomedicine, University Hospital Basel and University of Basel, Basel, Switzerland; National Institute of Environmental Health Sciences, UNITED STATES

## Abstract

The relationship between ABO blood groups (BG) and risk of incidence in cancers including gynecological cancers has been widely studied, showing increased incidence risk for BG A patients. As available data are inconsistent we investigated whether BG and their anti-glycan antibodies (anti-A and anti-B) have prognostic values in gynecological cancers. We retrospectively evaluated 974 patients with gynecological cancers in three cancer centers (Switzerland and Australia) between 1974 and 2014 regarding the relationships between clinico-pathological findings and the BG. Time to disease recurrence was significantly influenced by BG in patients with ovarian (n = 282) and vulvar (n = 67) cancer. BG O or B patients showed a significantly increased risk for ovarian cancer relapse compared to A, 59% and 82%, respectively (*p* = 0.045; HR O vs A = 1.59 (CI 1.01–2.51) and (*p* = 0.036; HR A vs B = 0.55 (CI 0.32–0.96). Median time to relapse for advanced stage (n = 126) ovarian cancer patients was 18.2 months for BG O and 32.2 for A (*p* = 0.031; HR O vs A = 2.07 (CI 1.07–4.02)). BG also significantly influenced relapse-free survival in patients with vulvar cancer (*p* = 0.002), with BG O tending to have increased relapse risk compared to A (*p* = 0.089). Blood groups hence associate with recurrence in ovarian and vulvar cancer: women with BG O seem to have a lower ovarian cancer incidence, however are more likely to relapse earlier. The significance of the BG status as a prognostic value is evident and may be helpful to oncologists in prognosticating disease outcome and selecting the appropriate therapy.

## Introduction

The ABO blood group (BG) in humans is the most important BG system in transfusion and transplantation medicine. It is defined by two glycans, antigen A (GalNAcα1-3(Fucα1–2)Galβ1) and B (Galα1-3(Fucα1–2)Galβ1) and is determined by the *ABO* gene that encodes the A and B allele, resulting in two different glycosyltransferase activities. These activities add either *N*-acetylgalactosamine or galactose to the precursor H antigen to form A or B antigen, respectively. The absence of both antigens in BG O owes to a frameshift mutation at the *N*-terminus of the enzyme [1]. ABO BG antigens are commonly expressed on cell surface glycosphingolipids or glycoproteins present on erythrocytes and on a variety of other human cells and tissues (e.g. gastro-intestinal, bronchopulmonary, skin and urogenital epithelial cells [2]), and also occur in various body fluids and secretions [3].

ABO BG are involved in several benign and malignant diseases [4] and the relationship between human BG and cancer is well known [5]. Several studies have shown associations between ABO BG and incidence and risk for various cancers [5] including ovarian cancer[5–7] and several plausible explanations have been proposed explaining the observed associations of ABO BG and cancer: these include inflammation, immune surveillance for malignant cells, modified expression of ABO BG antigens on cancer cells as a consequence of altered glycosyltransferase activities [8], intercellular adhesion and membrane signaling [9], single nucleotide polymorphisms and epigenetics [10].

Ovarian cancer (OC), usually diagnosed at an advanced FIGO stage, is the fifth leading cause of cancer death for women and the most lethal gynecological cancer in women and despite improved surgical techniques and drug regimens, overall survival has not changed significantly for several decades [11]. In addition, currently used screening methods seem not accurate enough: the combination of tumor marker CA125 and transvaginal ultrasound allows measurement of triaging indices such as the Risk of Malignancy Index (RMI) and facilitates discrimination between benign and malignant ovarian masses [12], and screening trials (PLCO-, UKCTOCS-trials) have not shown benefit in terms of disease specific survival [13, 14].

The search for new and highly specific biomarkers for both early disease detection and disease prognosis is still needed and ongoing. We have previously shown that the level of plasma-derived anti-glycan antibodies to P_1_ trisaccharide significantly discriminates between OC patients and healthy women, suggesting P_1_ as an OC-associated carbohydrate antigen[15]. Interestingly, P_1_ carbohydrate antigen belongs to the human P BG system and shares oligosaccharide sequences with P^k^ and P antigens [16]. In analogy to this and as we know that the classical blood system, with his anti-glycan antibodies, is involved in the pathogenesis of several malignancies, we were interested in looking for survival association in gynecological cancer liable to identify a prognostic marker.

Only a small body of data has been reported regarding associations between ABO BG and survival [17–20] and even fewer and inconsistent data are available for gynecological cancers in general and OC in particular [21, 22]. We therefore retrospectively evaluated and compared the clinic-pathological findings including relapse-free survival (RFS), disease-specific survival (DSS), and overall survival (OS) of a large gynecological cancer patient cohort (n = 974) to the ABO BG status.

## Materials and methods

### Study cohort description

Clinicopathological databases between 1974 and 2014 from three gynecological cancer centers in Switzerland (Basel, Zurich) and Australia (Sydney) were reviewed. Patients’ eligibility criteria included at least a histologically confirmed gynecological cancer, a complete remission after primary treatment, and an available BG status. Patients who deceased from causes other than cancer or developed a second primary tumor which is different from the cancer they had initially were censored in this study. Disease recurrence was diagnosed on a clinical basis (symptoms) and/or increasing tumor marker followed by radiological confirmation. Histological grades were defined according to the World Health Organization, and extent of disease by FIGO stage. All patients underwent surgery with curative intent, adjuvant chemotherapy and/or radiotherapy unless refused, as recommended by the interdisciplinary tumor conferences. These recommendations were based on international data and guidelines and were individualized depending to the patient’s co-morbidities. BG status (ABO system) of all patients was determined serologically before their surgery. Patients were followed up every 3 months for the first two years and then every 6 months until 5 years after completion of primary treatment, and then then once yearly. In total 974 patients were analyzed, subdivided into 282 cases of ovarian, 56 peritoneal, 23 tubal, 377 endometrial, 149 cervical, 11 vaginal, 67 vulvar, and 9 synchronous ovarian/endometrial cancers. This study was approved by the Swiss Medical Ethical Committee, EKNZ 2015–436. Neither written nor oral consent was necessary for thir retrospective study and data accession was anonymous.

### Statistical analysis

Descriptive statistics comparing the study groups are reported as counts and percentages or as mean and standard deviation (SD) as appropriate. Corresponding *p*-values were calculated using Fisher’s exact tests (counts) and t-Tests (ordinal data). Relationships between clinic-pathological findings, ABO BG, and outcome (RFS, DSS, and OS) were analyzed. RFS was defined as the period from the date of diagnosis to the date of disease recurrence (as described above). DSS was calculated from the date of diagnosis to death from disease. Deaths of unknown cause or other than disease were censored. Kaplan-Meier analysis was used to calculate the survival rate or time to event analysis (RFS, DSS, and OS) with a 95% confidence interval (CI). Data for 5-year OS were also reported. The Log-rank test was used to compare the survival curves. Additionally, Cox-Regression analysis including a center effect comparing each blood group was performed. Analyses were not adjusted for covariates. Results (median values) are reported with hazard ratios (HR), corresponding 95% CI’s and *p*-values. Reported *p*-values were two-sided and *p* < 0.05 was considered statistically significant. The statistical analyses were performed using R version 3.0.1.

## Results

### Clinico-pathological characteristics for all gynecological cancers patients sorted by blood groups

This study includes 974 patients with various gynecological cancers. The mean age was 62.8 ± 13.7 years and the mean follow-up 4.8 ± 33.7 years. The BG distribution was 471 patients with BG A (48.4%), 94 patients with B (9.6%), 375 patients with O (38.5%), 34 patients with AB (3.5%), and was similar to that of the general population in Europe. The cohort comprised ovarian (n = 282), peritoneal (n = 56), tubal (n = 23), cervical (n = 149), endometrial (n = 377), vaginal (n = 11), and vulvar cancer (n = 67) patients. This and additional information on the study cohort including tumor type, histology, stage, grade, residual disease, survival status, and recurrence status regarding the BG status is summarized in Table 1.

**Table 1:** Clinico-pathological data of gynecological cancer cohort sorted by blood group.

	ABO blood group						
	ALL	O	A	AB	B	*p*	n
Characteristic							
**Number of women**	974	375	471	34	94		
**Percentage of total (%)**	100.0	38.5	48.4	3.5	9.7		
**Cancer Center**						0.038	974
Basel	708 (72.7%)	265 (70.7%)	364 (77.3%)	22 (64.7%)	57 (60.6%)		
Sydney	210 (21.6%)	89 (23.7%)	83 (17.6%)	9 (26.5%)	29 (30.9%)		
Zürich	56 (5.75%)	21 (5.60%)	24 (5.10%)	3 (8.82%)	8 (8.51%)		
**Mean age (years) (±SD)**	62.8 (13.7)	63.1 (13.8)	62.9 (13.5)	62.3 (12.2)	61.5 (14.5)	0.786	940
**Organ**						0.035	974
Cervix	149 (15.3%)	54 (14.4%)	81 (17.2%)	5 (14.7%)	9 (9.57%)		
Endometrium	377 (38.7%)	156 (41.6%)	182 (38.6%)	13 (38.2%)	26 (27.7%)		
Ovaries	282 (29.0%)	100 (26.7%)	138 (29.3%)	10 (29.4%)	34 (36.2%)		
Ovaries & Endometrium	9 (0.92%)	2 (0.53%)	5 (1.06%)	1 (2.94%)	1 (1.06%)		
Peritoneum	56 (5.75%)	22 (5.87%)	18 (3.82%)	3 (8.82%)	13 (13.8%)		
Fallopian tube	23 (2.36%)	9 (2.40%)	9 (1.91%)	1 (2.94%)	4 (4.26%)		
Vagina	11 (1.13%)	8 (2.13%)	3 (0.64%)	0 (0.00%)	0 (0.00%)		
Vulva	67 (6.88%)	24 (6.40%)	35 (7.43%)	1 (2.94%)	7 (7.45%)		
**Tumour type**						0.183	974
Adenocarcinoma	733 (75.3%)	286 (76.3%)	345 (73.2%)	25 (73.5%)	77 (81.9%)		
Squamous cell carcinoma	193 (19.8%)	71 (18.9%)	103 (21.9%)	6 (17.6%)	13 (13.8%)		
Carcinosarcoma (MMMT)	26 (2.67%)	11 (2.93%)	12 (2.55%)	1 (2.94%)	2 (2.13%)		
Adenosarcoma	1 (0.10%)	1 (0.27%)	0 (0.00%)	0 (0.00%)	0 (0.00%)		
Adenosquamous carcinoma	2 (0.21%)	1 (0.27%)	1 (0.21%)	0 (0.00%)	0 (0.00%)		
Sarcoma	7 (0.72%)	4 (1.07%)	0 (0.00%)	2 (5.88%)	1 (1.06%)		
Carcinoid	1 (0.10%)	1 (0.27%)	0 (0.00%)	0 (0.00%)	0 (0.00%)		
Brenner tumor	3 (0.31%)	0 (0.00%)	2 (0.42%)	0 (0.00%)	1 (1.06%)		
Sertoli-Leydig tumor	2 (0.21%)	0 (0.00%)	2 (0.42%)	0 (0.00%)	0 (0.00%)		
Granulosacell tumor	6 (0.62%)	0 (0.00%)	6 (1.27%)	0 (0.00%)	0 (0.00%)		
**Histology**						0.427	709
Serous	251 (35.4%)	92 (33.3%)	115 (34.3%)	9 (37.5%)	35 (47.3%)		
Endometrioid	339 (47.8%)	132 (47.8%)	165 (49.3%)	11 (45.8%)	31 (41.9%)		
Mucinous	24 (3.39%)	9 (3.26%)	10 (2.99%)	1 (4.17%)	4 (5.41%)		
Clear cell	23 (3.24%)	9 (3.26%)	12 (3.58%)	2 (8.33%)	0 (0.00%)		
Neuroendocrine	3 (0.42%)	1 (0.36%)	1 (0.30%)	0 (0.00%)	1 (1.35%)		
Mixed/unknown/other	69 (9.73%)	33 (4.65%)	32 (4.51%)	1 (0.14%)	3 (0.42%)		
**FIGO Stage**						0.524	662
I	259 (39.1%)	105 (40.5%)	111 (36.2%)	11 (45.8%)	32 (44.4%)		
II	85 (12.8%)	31 (12.0%)	45 (14.7%)	4 (16.7%)	5 (6.94%)		
III	242 (36.6%)	95 (36.7%)	110 (35.8%)	7 (29.2%)	30 (41.7%)		
IV	76 (11.5%)	28 (10.8%)	41 (13.4%)	2 (8.33%)	5 (6.94%)		
**Tumour grade**						0.510	762
G1	185 (24.3%)	66 (22.2%)	96 (26.2%)	3 (12.5%)	20 (26.7%)		
G2	238 (31.2%)	98 (33.0%)	112 (30.6%)	6 (25.0%)	22 (29.3%)		
G3	339 (44.5%)	133 (44.8%)	158 (43.2%)	15 (62.5%)	33 (44.0%)		
**Residual Disease**						0.571	441
optimal debulking	303 (68.7%)	118 (67.4%)	144 (70.9%)	10 (76.9%)	31 (62.0%)		
suboptimal debulking	138 (31.3%)	57 (32.6%)	59 (29.1%)	3 (23.1%)	19 (38.0%)		
**Survival status**						0.962	933
alive	829 (88.9%)	317 (89.0%)	401 (88.5%)	29 (87.9%)	82 (90.1%)		
dead of disease	104 (11.1%)	39 (11.0%)	52 (11.5%)	4 (12.1%)	9 (9.89%)		
**Recurrence**						0.009	974
no	750 (77.0%)	298 (79.5%)	368 (78.1%)	24 (70.6%)	60 (63.8%)		
yes	108 (23.0%)	77 (20.5%)	103 (21.9%)	10 (29.4%)	34 (36.2%)		

Data from gynecological cancer centers (Basel and Zurich, Switzerland) and Sydney (Australia) collected between 1974 and 2014. *P*-values calculated by t-test or Fisher’s exact tests.

### Effect of blood group status on RFS, DSS, and OS in all gynecological cancers patients

Time-to-event analysis was performed for all 7 gynecological cancer types. For DSS, no significant associations to BG were found for these cancer types: ovarian (*p =* 0.696), peritoneal (*p* = 0.28), tubal (*p* = 0.366), cervical (*p* = 0.723), endometrial (*p* = 0.39), vaginal (*p* = 0.26), and vulvar (VC, *p* = 0.29) cancer. No significant associations to BG were also found for RFS in peritoneal (*p* = 0.889), tubal (*p* = 0.814), cervical (*p* = 0.638), endometrial (*p* = 0.492) or vaginal (*p* = 0.480) cancer. In contrast, associations for RFS and BG were found for OC and VC. No significant associations for OS were found for the whole cohort (*p* = 0.287).

### Effect of blood group status on RFS and OS in ovarian cancer patients

The OC group comprised 282 patients with a mean age at diagnosis of 60.7 ± 13.7 years and mean follow-up time of 3.26 ± 4.12 years. BG distribution was 100 (35.5%) with BG O, 138 (48.9%) with A, 10 (3.5%) with AB, and 34 (12.1%) with B. These and additional clinico-pathological data are given in Table 2.

**Table 2:** Clinico-pathological data of ovarian cancer cohort sorted by blood group.

	ABO blood group						
	ALL	O	A	AB	B	*p*	n
Characteristic							
**Number of women**	282	100	138	10	34		282
**Percentage of total (%)**	100	35.46	48.94	3.55	12.06		
**Cancer Center**	** **					0.16	282
Basel	160 (56.7%)	55 (55.0%)	85 (61.6%)	6 (60.0%)	14 (41.2%)		
Sydney	87 (30.9%)	36 (36.0%)	33 (23.9%)	3 (30.0%)	15 (44.1%)		
Zürich	35 (12.4%)	9 (9.00%)	20 (14.5%)	1 (10.0%)	5 (14.7%)		
**Mean age (years) (±SD)**	60.7 (13.7)	61.1(13.9)	60.2 (13.3)	57.4 (12.3)	63.1 (15.1)	0.599	276
**Tumour type**	** **					0.397	282
Adenocarcinoma	257 (91.1%)	93 (93.0%)	123 (89.1%)	10 (100%)	31 (91.2%)		
Carcinosarcoma (MMMT)	13 (4.61%)	6 (6.00%)	5 (3.62%)	0 (0.00%)	2 (5.88%)		
Carcinoid	1 (0.35%)	1 (1.00%)	0 (0.00%)	0 (0.00%)	0 (0.00%)		
Brenner tumor	3 (1.06%)	0 (0.00%)	2 (1.45%)	0 (0.00%)	1 (2.94%)		
Sertoli-Leydig tumor	2 (0.71%)	0 (0.00%)	2 (1.45%)	0 (0.00%)	0 (0.00%)		
Granulosacell tumor	6 (2.13%)	0 (0.00%)	6 (4.35%)	0 (0.00%)	0 (0.00%)		
**Histology**	** **					0.645	261
Serous	160 (61.3%)	53 (56.4%)	83 (65.9%)	4 (40.0%)	20 (64.5%)		
Endometrioid	42 (16.1%)	16 (17.0%)	18 (14.3%)	2 (20.0%)	6 (19.4%)		
Mucinous	21 (8.05%)	8 (8.51%)	8 (6.35%)	1 (10.0%)	4 (12.9%)		
Clear cell	11 (4.21%)	5 (5.32%)	4 (3.17%)	2 (20.0%)	0 (0.00%)		
Neuroendocrine	1 (0.38%)	1 (1.06%)	0 (0.00%)	0 (0.00%)	0 (0.00%)		
Mixed/unknown/other	26 (9.96%)	11 (11.70%)	13 (10.32%)	1 (10.0%)	1 (3.23%)		
**FIGO Stage**	** **					0.59	247
I	64 (25.9%)	22 (23.9%)	27 (23.7%)	4 (40.0%)	11 (35.5%)		
II	21 (8.50%)	10 (10.9%)	8 (7.02%)	1 (10.0%)	2 (6.45%)		
III	126 (51.0%)	44 (47.8%)	61 (53.5%)	4 (40.0%)	17 (54.8%)		
IV	36 (14.6%)	16 (17.4%)	18 (15.8%)	1 (10.0%)	1 (3.23%)		
**Tumour grade**	** **					0.519	216
G1	34 (15.7%)	14 (17.5%)	14 (13.7%)	1 (12.5%)	5 (19.2%)		
G2	35 (16.2%)	17 (21.2%)	12 (11.8%)	1 (12.5%)	5 (19.2%)		
G3	147 (68.1%)	49 (61.3%)	76 (74.5%)	6 (75.0%)	16 (61.5%)		
**Residual Disease**	** **					0.783	170
optimal debulking	106 (62.4%)	41 (61.2%)	47 (62.7%)	5 (83.3%)	13 (59.1%)		
suboptimal debulking	64 (37.6%)	26 (38.8%)	28 (37.3%)	1 (16.7%)	9 (40.9%)		
**Survival status**						0.781	274
alive	226 (82.5%)	81 (82.7%)	107 (80.5%)	9 (90.0%)	29 (87.9%)		
dead of disease	48 (17.5%)	17 (17.3%)	26 (19.5%)	1 (10.0%)	4 (12.1%)		
**Recurrence**						0.128	282
no	174 (61.7%)	67 (67.0%)	86 (62.3%)	6 (60.0%)	15 (44.1%)		
yes	108 (38.3%)	33 (33.0%)	52 (37.7%)	4 (40.0%)	19 (55.9%)		

Data from gynecological cancer centers (Basel and Zürich, Switzerland) and Sydney (Australia) collected between 1974 and 2014. *P*-values calculated by T-Tests or Fisher’s exact Tests.

**Fig 1:**
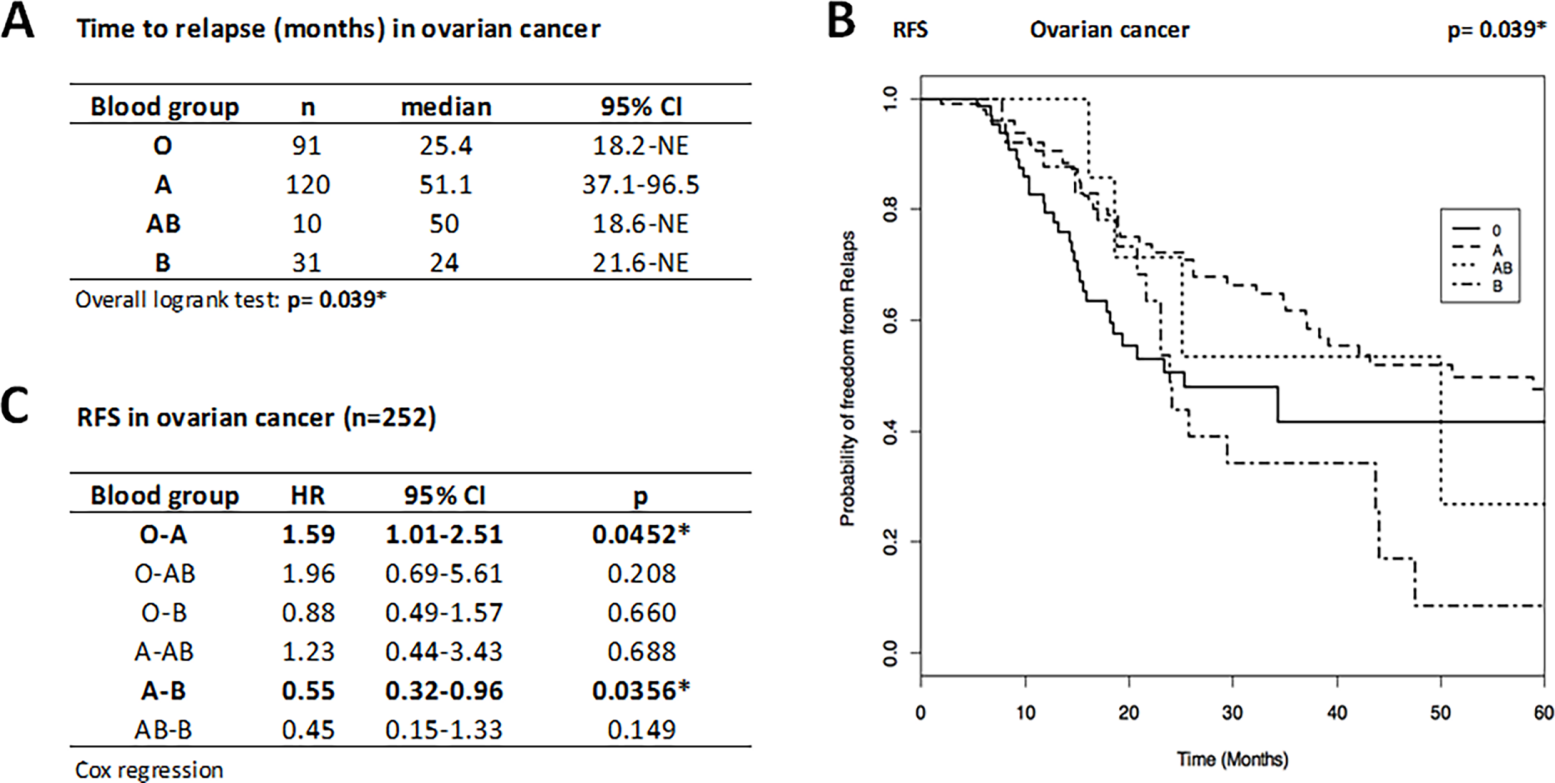
**Time to relapse (A), Kaplan-Meier curve for RFS (B), and HR for disease recurrence (C) in ovarian cancer patients (n = 252)**. BG O and B patients showed a significantly increased risk for relapse compared to A patients (59%, *p* = 0.045 and 82%, *p* = 0.036, respectively; Cox regression). Hence, BG A patients have better prognosis with a significant longer RFS than those with O and B. Time to relapse presented as median (months) and 95%CI and compared by overall logrank test and disease recurrence risk presented as HR and 95%CI. Statistical significance marked by asterisks (*) or highlighted. NE, not estimable. RFS given as probability of freedom from relapse as a function of time (months).

The median time until relapse for OC patients was 25.4 months for BG O, 51.1 months for A, 50.0 months for AB and 24.0 months for B (Fig 1A and 1B), indicating that patients with BG A relapsed about two years later than patients with BG O.

Statistical analysis showed a significant (*p* = 0.039) overall influence of the BG on the time to relapse in OC (Fig 1C): patients with BG O showed a statistically significant (*p* = 0.045) 59% (HR O vs A = 1.59) increased risk for OC relapse and patients with BG B a statistically significant (*p* = 0.036) 82% (HR B vs A = 1.82 = 1/0.55 given in Fig 1C) increased risk for OC relapse compared to those with BG A, indicating that patients with BG A have better prognosis with a significant longer relapse-free survival than those with BG O and BG B. The other comparisons did not reveal any significant differences for relapse risk. Likewise, no difference in OS was found among the blood groups (*p* = 0.665). The 5-year OS was 0.694 (95%CI: 0.531–0.907) for BG O and 0.734 (95%CI: 0.624–0.863) for BG A (values for BG AB and BG B not reported because of low number of cases and events).

### Effect of blood group status on RFS and OS in vulvar cancer patients

**Fig 2:**
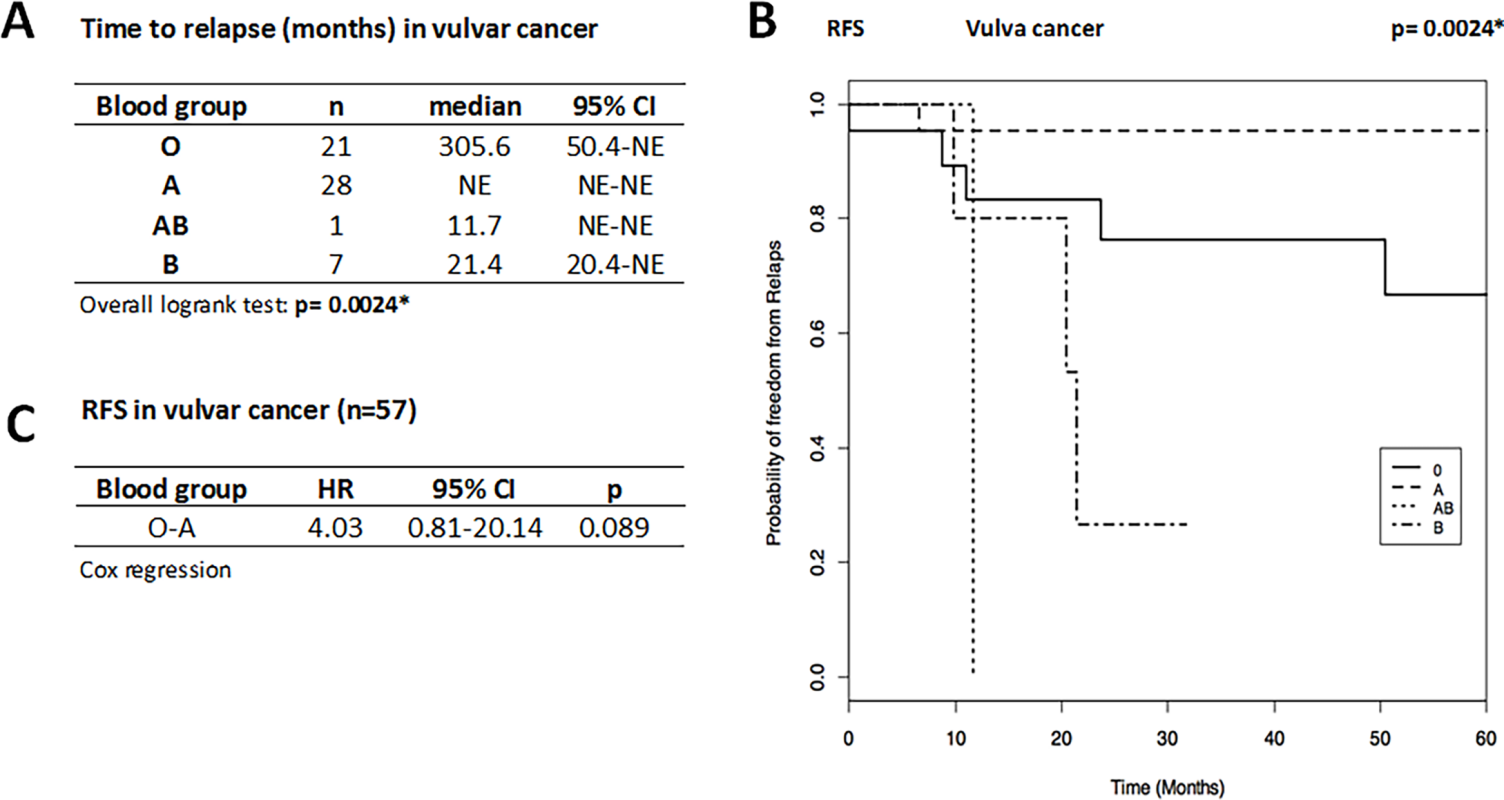
**Time to relapse (A), Kaplan-Meier curve for RFS (B), and HR for disease recurrence (C) in vulvar cancer patients (n = 57)**. BG O patients tend to have increased risk for relapse compared to A patients A (HR O vs A = 4.03, 95%CI: 0.81–20.14, *p* = 0.089), i.e. A patients have better prognosis with a trend to longer RFS than patients with BG O. Time to relapse presented as median (months) and 95%CI and compared by overall Logrank Test. Disease recurrence risk presented as HR and 95%CI by Cox regression (only possible for O vs A owing the small sample size for AB and B). Statistical significance marked by asterisks (*) or highlighted. NE, not estimable. RFS given as probability of freedom from relapse as a function of time (months).

The group with VC comprised 67 patients with a mean age at diagnosis of 70.0 ± 13.8 years and mean follow-up time of 5.15 ± 5.5 years. BG distribution was 35.5% with BG O, 52.2% with A, 1.5% with AB, and 10.5% with B. The data showed (Fig 2A and 2B) that the median time until relapse was 305.6 months for BG O (n = 21), 21.4 months for BG B (n = 7), 11.7 months for the one patient with BG AB, and was not estimable for the 28 patients with BG A (none relapsed within 60 months). Despite the low number of cases and non-estimable data, a highly significant (*p* = 0.0024) relationship between the BG and the time to relapse for VC was found. Cox regression (Fig 2C) analysis for patients with BG O compared to A showed a trend for a 4-times longer time to relapse for BG A patients, whereas comparisons among the other groups were obsolete because of respectively low number of cases and non-estimable data. Data for OS are not reported due to low number of cases and events.

Taken together, the data indicate that BG O is associated with higher risk for disease recurrence in OC and VC.

### Effect of blood group status on FIGO III ovarian adenocarcinoma

**Fig 3:**
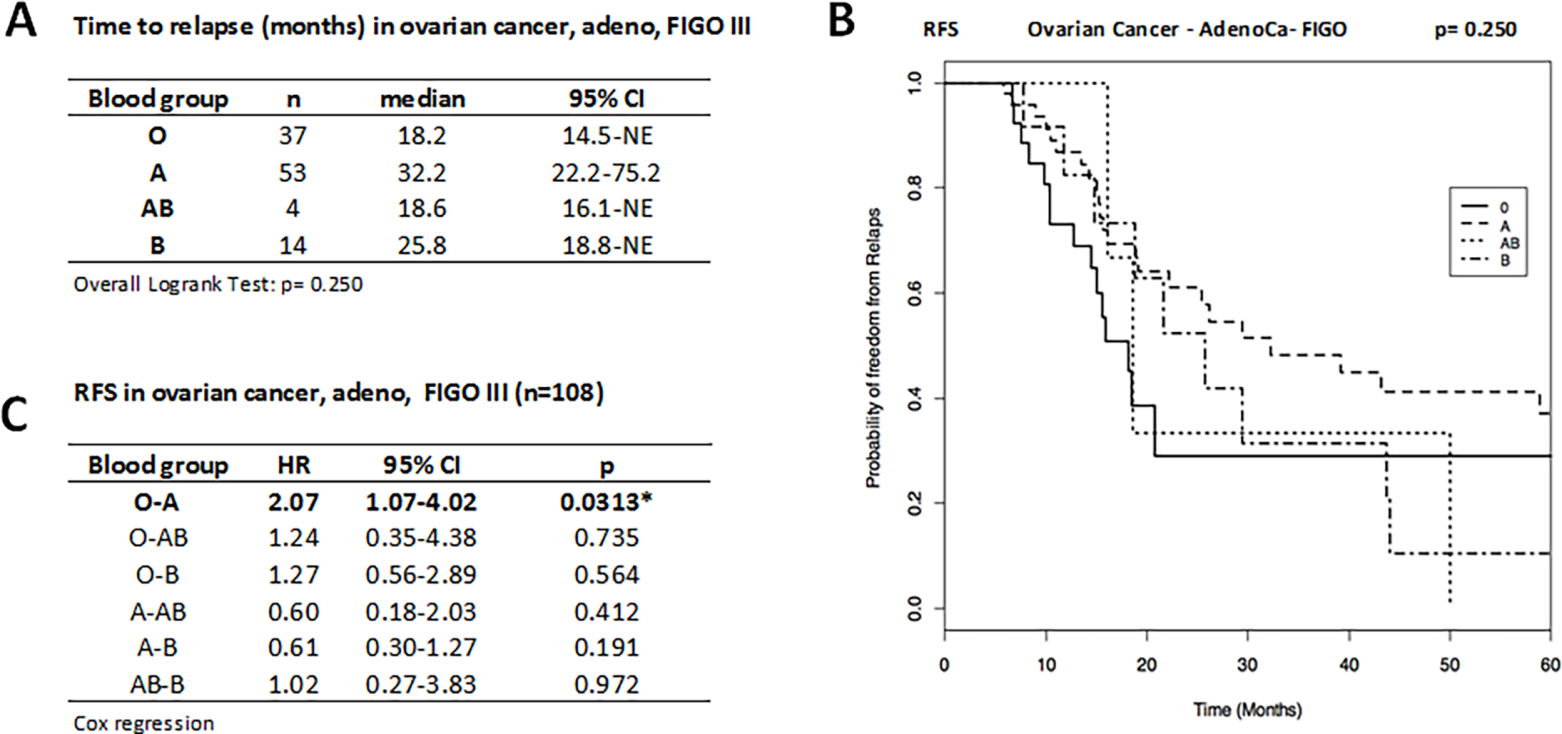
**Time to relapse (A), Kaplan-Meier curve for RFS (B), and HR for disease recurrence (C) in the FIGO III adenocarcinoma patient subgroup (n = 108)**. BG O patients have two time increased risk for relapse compared to A patients A, i.e. BG A patients have better prognosis with a significant longer RFS than patients with O. Time to relapse presented as median (months) and 95%CI and compared by overall Logrank test. Disease recurrence risk presented as HR and 95%CI by Cox regression. Statistical significance marked by asterisks (*) or highlighted. NE, not estimable. RFS given as probability of freedom from relapse as a function of time (months).

In order to eliminate the contribution of the prognostic effect of the FIGO stage we determined the RFS and OS in FIGO stage III ovarian adenocarcinoma patients. In this more homogenous subgroup (n = 108), the median time until relapse was 18.2 months for BG O (n = 37), 32.2 months for A (n = 53), 18.6 months for AB (n = 4), and 14 months for B (n = 14), and hence were not significantly different among each other (Fig 3A and 3B). Cox regression analysis showed a statistically significant two time increased recurrence risk for FIGO III ovarian carcinoma patients with BG O compared to BG A (HR O vs A = 2.07; 95%CI: 1.07–4.02; *p* = 0.0313), while other comparisons did not reveal any significant differences (Fig 3B). The OS did not differ among the blood groups (p = 0.115). The 5-year OS was 0.714 (95%CI: 0.478–1.000) for BG O and 0.789 (95%CI: 0.619–1.000) for BG A (data for BG AB and BG B not reported due to low number of cases and events).

## Discussion

This retrospective study with various gynecological malignancies showed that relapse-free survival (RFS) in OC and VC (but not on other gynecological cancers such as peritoneal, cervical, endometrial, tubal, and endometrial cancers) was significantly influenced the ABO BG status. Specifically, BG O and B ovarian cancer patients had a considerably increased risk for recurrence compared to BG A patients (i.e. BG A patients have a marked better prognosis with a longer time-to-relapse than BG O and B patients) and BG A vulvar cancer patients at least tend to have a longer time-to-relapse than patients with BG O. No influence was observed for DSS and OS for both cancers. Our data indicate that RFS in OC and VC patients is associated with the ABO blood type, suggesting that the ABO status is an important factor for RFS. The present study is the first to show the prognostic value of the ABO BG status for RFS, in particular BG A, in OC and VC and is also the largest data in the English literature addressing the prognostic value of ABO BG in regards to RFS and DSS in gynecological cancers.

Very little is known from the literature about the relationship between ABO BG and cancer prognosis (summarized in Table 3). One study on gynecological cancers published (in Italian) in 1995 [21] reported negative associations between overall survival (OS) and BG A in endometrial and OC when compared to BG O, and a positive association between BG A and OS in cervical cancer patients. Considering this it seems that OC patients with BG A have a lower risk for recurrence, no different DSS, but a worse OS than BG O patients, but opposed results were reported very recently in study with over 700 patients, demonstrating a significantly better OS in OC patients with BG A compared to BG O or non-A [22]. In our study, however, no difference in OS was found in OC patients for any BG.

**Table 3:** Studies on the prognostic value of ABO blood group by cancer type.

Author	Year	n	Cancer	Blood group	Influence on prognostic data		Country	Publication
					negative/positive	Survival data		
								
Kaffenberger	2012	900	RCC	non-O	negative	OS	USA	BJU international 2012;110: E641-6
de Martino	2014	556	RCC	no association			Austria	BJU international 2014;113: E62-6
Lee	2015	3'172	RCC	no association			Korea	J Cancer Res Clin Oncol
Unal	2013	81	NSCLC	no association			Turkey	APJCP 2013;14: 3945–8
Fukumoto	2015	333	NSCLC	A, AB	negative	DFS, OS	Japan	Journal of epidemiology 2015;25: 110–6.
Yang	2014	496	ESCC	non-O	positive	OS	China	Int J Clin Exp Med 2014;7: 2214–8
Qin	2015	548	ESCC > subgroup with negativ NL	non-AB	positive	OS	China	OncoTargets and therapy 2015;**8**: 947–53
Xu	2016	1'412	Gastric	AB	positive	OS	China	J Surg Res 2016;201: 188–95.
			> subgroup after Gastrectomy	A	negative	OS		
Dandona	2010	417	Pancreas	no association			USA	J Natl Cancer Inst 2010;102: 135–7
Ben	2011	1'431	Pancreas	no association			China	Int J Cancer 011;128: 1179–86
		316	> subgroup with curative resection	O	positive	OS		
Rahbari	2012	627	Pancreas	O	positive	OS	Germany	BMC cancer 2012;12: 319
Cao	2014	1'555	Colon	AB	positive	OS	China	British journal of cancer 2014;111: 174–80
Holdsworth	1985	1'001	Breast	O	positive	DFS	GB	Br Med J 1985;290: 671–3
Costantini	1990	315	Breast	O	positive	OS	Italy	Oncology 1990;47: 308–12
Klimant	2011	426	Breast	no association			USA	Clinical medicine & research 2011;9: 111–8
Gates	2012	2'036	Breast	no association			USA	Int J Cancer 2012;130: 2129–37
Cihan	2014	335	Breast	A, O	positive	DFS, OS	Turkey	APJCP 2014;15: 4055–60
Marinaccio	1995	92	Ovary	A	negative	OS	Italy	Minerva ginecologica 1995;47: 69–76
		237	Endometrium	A	negative	OS		
		639	Cervix	no association				
Cozzi	2017	713	Ovary		positive	OS	USA	PLoS One 2017; 30;12 (5):e0178965
*Montavon*	*2018*	*282*	*Ovary*	*A*	*positive*	*RFS*	*Swiss*, *AUS*	
* *	* *	* *	* *	* *	*no association*	*OS*	* *	
* *	* *	*56*	*Peritonum*	*no association*			* *	
* *	* *	*23*	*Fallopian tube*	*no association*			* *	
* *	* *	*377*	*Endometrium*	*no association*			* *	
* *	* *	*149*	*Cervix*	*no association*			* *	
* *	* *	*67*	*Vulva*	*A*	*positiv*	*RFS*	* *	
* *	* *	* *	* *	* *	*no association*	*OS*	* *	
* *	* *	*11*	*Vagina*	*no association*			* *	

RCC (renal cell carcinoma), NSCLC (Non-small Cell Lung Cancer), ESCC (oesophageal squamous cell carcinoma), OS (overall survival), DFS (Disease Free Survival), RFS (Relapse Free Survival). Current study in italic.

The largest body of data available however relates to the association of ABO BG with cancer risk and incidence. The first indication of a possible relationship between BG and cancer risk was published in 1953, reporting a 20% increased incidence of gastric cancer in BG A compared to O [23]. Since then an increasing numbers of often inconsistent data were published, suggesting that the biological role of ABO antigens may be disease-specific. Patients with BG A and AB have an increased risk of gallbladder [24], and nasopharyngeal carcinomas [25], whereas non-O female blood group carriers have been identified with a higher risk of developing renal cell cancer [26]. BG B has been significantly associated with cardiac and oesophageal carcinomas [27, 28]. BG O carriers have a reduced risk of developing basal cell carcinoma, squamous cell carcinoma of the skin [29], and a lower risk of pancreatic cancer [30]. Other associations have been reported, but the data are not reproducible for lung [31, 32], breast [33, 34] and colorectal cancers [35, 36]. A recent meta-analysis of 89 eligible studies with 100’554 cases from 30 cancer sites calculated a pooled OR for overall cancer risk of 1.12 for A vs non-A groups and 0.84 for O vs non-O groups [5]. Reports for gynecological cancers are also rare and mainly suggest increased OC risk for BG A compared to non-A and a decreased risk for BG O [5–7]. No significant association of blood groups with cervical cancer risk were reported in 8 studies [7] and two studies reported no significant associations in endometrial cancer [7]. Other gynecological cancers are underreported with only two studies without any significant difference in invasive squamous cell carcinoma of the vulva [37, 38].

All these studies underline the biological role of the BG regarding incidence risk, prognosis, and outcome in malignant diseases including ovarian, vulvar, and endometrial cancer. Associations between ABO blood type and modified immune response have been described [39, 40], but this may not fully explain how the ABO BG are linked to cancer risk incidence. Welshinger *et al*. have shown that, although the ovarian surface epithelium usually does not express BG antigens, ABO antigens were expressed in some areas of activated surface epithelium, in inclusion cysts and also by one-half of ovarian carcinomas [41], but it is still poorly understood how BG antigens or altered abundance of BG antigens influence carcinogenesis and whether BG antigen expression is a consequence of malignant transformation.

It is also poorly understood how BG antigens relate to disease outcome and prognosis. The expression of BG antigens on cancer cells can be subject to genetic and epigenetic modifications, e.g. ABO promotor methylation, which in turn might be related to tumor invasion and metastasis [10]. Certain tumor antigens may mimic the structure of antigens of the ABO system. Forssmann antigen, predominantly expressed in stomach and colon tumors, is structurally almost identical to A antigen determinant: BG A carriers may have a diminished tumor immune response owing the reduced ability in recognizing and attacking tumor cells expressing Forssmann antigen[5]. Abundance of A and B antigen in carcinomas derived from tissues normally not expressing these antigens may promote cancer aggressiveness by increasing cell motility, resistance to apoptosis, and immune escape [2].

The most important finding of the present study is that OC patients and to some extent vulvar cancer patients with BG A have substantially longer RFS compared to BG O (and B). This finding is in line with the recently reported longer survival for OC patients with BG A compared to BG O or non-A [22] and suggests that the lower recurrence risk accounts for this better survival. This finding presents a novelty regarding outcome and ABO BG in gynecological cancers. But open questions remain such as why BG A patients have a better RFS prognosis, why this advantage is not reflected for DSS, and why no association was found for other gynecological cancers. At least our data suggest that abundance of A antigen (or anti-B antibodies) delays recurrence or that B antigen (or anti-A antibodies) promotes recurrence in these cancers, but it is unknown by which mechanisms these immune molecules influence disease recurrence. Likewise intriguing is the observed reciprocal relationship between incidence risk and RFS in OC: women with BG O seem to have a lower incidence, however are more likely to relapse earlier.

Despite the strong indication of a positive role of BG A on prolonged RFS in OC, we recognizes that the occasionally small sample number (despite an almost 1000 patient mighty cohort) may limit the value of statistical analysis e.g. for AB patients (least frequent blood group), rare gynecological cancers (e.g. vulvar, vagina), and small subgroups (e.g. histology, stage, grade), warranting the conduction of studies with larger cohorts.

## Abbreviations

BG: blood group
CA125: cancer antigen 125
CI: confidence interval
DSS: disease-free survival
FIGO: International Federation of Gynecology and Obstetrics
HR: Hazard ratio
OC: ovarian cancer
OS: overall survival
RFS: relapse-free survival
RMI: risk of malignancy index
VC: vulvar cancer
